# The Relationship Between Sleep Disorders and Combination of Diabetes and Sarcopenia in Adults Aged 45 Years or Older: 10-Year Nationwide Prospective Cohort Study

**DOI:** 10.2196/66372

**Published:** 2025-06-02

**Authors:** Shugang Li, Yimi Wang, Linzhi Li, Hao Wu

**Affiliations:** 1 School of General Practice and Continuing Education, Capital Medical University, No.10 Xitoutiao, Youanmenwai, Fengtai District, Beijing, 100069, China, 86 13366003306, 86 83911713; 2 School of Medicine, Shihezi University, Shihezi, China

**Keywords:** diabetes, sarcopenia, combined effect, sleep disorders, elderly, cohort study

## Abstract

**Background:**

With changes in lifestyle, the issue of sleep disorders is becoming increasingly common. Diabetes and sarcopenia have been found to be independently associated with sleep disorders. However, fewer studies have explored the interaction between the combination of diabetes and sarcopenia at different stages and sleep disorders.

**Objective:**

This study aimed to explore the relationship between the combination of diabetes and sarcopenia and the incidence of sleep disorders in adults aged 45 years and older.

**Methods:**

Based on data from the CHARLS (China Health and Retirement Longitudinal Study), we selected participants with comprehensive diagnostic information on diabetes and sarcopenia from 2011 who had normal sleep duration at baseline and checked their follow-up information of sleep duration from 2013, 2015, 2018, and 2020. Diabetes was classified into diabetes (D), prediabetes (PD), and nondiabetes (ND), and sarcopenia was divided into sarcopenia (S), possible sarcopenia (PS), and nonsarcopenia (NS). The participants were divided into DS, DPS, DNS, PDS, PDPS, PDNS, NDS, NDPS, and NDNS groups. Kaplan-Meier survival curves, the log-rank test, Cox proportional hazards regression, and restricted cubic spline models were used for statistical analysis.

**Results:**

A total of 4936 participants were included in this study. The DS group had the highest incidence of sleep disorders: 49.32%, 28.57%, 36.36%, and 80.00% in 2013, 2015, 2018, and 2020 respectively. In the crude model, compared with the NDNS group, the risk of sleep disorders was increased in the DS group (hazard ratio [HR] 1.707, 95% CI 1.196‐2.437), PDS (HR 1.599, 95% CI 1.235‐2.071), NDS (HR 1.465, 95% CI 1.282‐1.674), and DPS group (HR 1.318, 95% CI 1.097‐1.583). The risk was increased but not statistically significant in the PDPS group (HR 1.160, 95% CI 0.987‐1.365). After adjusting for covariates, the risk of sleep disorders remained statistically significant in the DS group (HR 1.515, 95% CI 1.059‐2.167) and was significantly higher in the PDS (HR 1.423, 95% CI 1.096‐1.847) and NDS (HR 1.279, 95% CI 1.113‐1.468) groups than that in the NDNS group. The nonlinear associations between appendicular skeletal muscle mass, grip strength, 5-time chair test, fasting plasma glucose, and sleep disorders were observed and described.

**Conclusions:**

The combination of diabetes and sarcopenia significantly increases the risk of sleep disorders in adults aged 45 years and older. and the implementation of progression control of both diabetes and sarcopenia may be helpful to prevent sleep disorders in this population.

## Introduction

With changes in lifestyle, the issue of sleep disorders is becoming increasingly common. Studies report the worldwide prevalence of obstructive sleep apnea, poor sleep quality, other sleep problems, insomnia, and excessive daytime sleepiness to be 46%, 10%, 37%, 29%, and 19% respectively [1]. Even among adolescents, the prevalence of short and disturbed sleep is as high as 34.1% [2]. The impact of sleep disorders on the health of the older adults is more obvious, which can lead to cardiovascular disease [3], dementia [4], depression, and many other health problems. With the acceleration of aging, sleep disorders in older adults has become a global public health problem. A large number of studies have focused on the factors influencing sleep disorders. In addition to psychological [5], environmental [6], lifestyle [7] factors, chronic diseases, such as diabetes [8] and sarcopenia [9] are found to be closely related to sleep disorders. According to the International Diabetes Federation report, a total of 537 million adults worldwide had diabetes in 2021, which is expected to increase by 46% to 783 million by 2045 [10]. The prevalence of diabetes in people aged ≥45 years in China is 10.6% [11], and there are significant differences between men and women, and individuals in urban and rural areas. With the increase in age, human function significantly declines, and coupled with the impact of metabolic diseases, sarcopenia is becoming more common in older adults. With a reported prevalence of 27% in individuals over 60 years of age [12], sarcopenia has become one of the common comorbidities in older adults.

Diabetes is an important factor affecting sleep disorders [13]. Studies have found a significant association between obstructive sleep apnea risk and type 2 diabetes (odds ratio [OR] 2.44, 95% CI 1.78-3.35; *P*<.001) [14]. Compared with patients without diabetes, both patients with type 1 (hazard ratio [HR] 1.41, 95% CI 1.37-1.46, ) and type 2 diabetes (HR 1.40, 95% CI 1.37‐1.44) have a significantly increased risk of sleep disorders [15]. In addition, studies have shown that sarcopenia is a chronic disease that induces sleep disorders [16], and people with both sarcopenia and diabetes have a significantly increased risk of sleep abnormalities [17]. Even self-reported sleep duration may provide the possibility for early prediction of sarcopenia. These findings suggest a complicated relationship between diabetes, sarcopenia, and sleep disorders.

However, these existing studies have not elucidated the risk of sleep disorders in populations with comorbid diabetes and sarcopenia; most studies have analyzed the relationships between diabetes and sleep disorders, as well as the impact of sarcopenia on sleep disorders. Another important issue is that both diabetes guidelines and the expert consensus on sarcopenia emphasize prediabetes and possible sarcopenia, which are similar to large icebergs beneath the surface—and their contribution to sleep disorders also warrants attention. With aging, the prevalence of diabetes and sarcopenia gradually increases in the older population, which prompts us to consider whether an interaction between diabetes and sarcopenia at different stages significantly increases the risk of sleep disorders. Previous studies have mostly explored the relationship between sarcopenia, diabetes, and sleep disorders using a cross-sectional design. On the one hand, there is a lack of evidence from longitudinal cohort designs; on the other hand, the interaction of the risk between diabetes and sarcopenia at different stages and sleep disorders has not been elucidated. Based on this scientific question, the present study used the China Health and Retirement Longitudinal Study (CHARLS) to analyze the impact of the interaction of diabetes and sarcopenia on the occurrence of sleep disorders over four longitudinal follow-up visits (ie, 2013, 2015, 2018, 2020) from the 2011 baseline population, to provide a reference for the prevention and treatment of sleep disorders in adults aged ≥45 years.

## Methods

### Participants and Study Design

The participants for this study were derived from the CHARLS, a nationally representative longitudinal survey initiated in 2011. It covered 150 county-level units, 450 village-level units, and 10,257 households across 28 provinces. These participants were followed up every 2 to 3 years. CHARLS covers basic personal information, household structure, health status, physical measurements, health service utilization, health insurance, and basic community characteristics. Four waves of follow-up data collection have been completed so far, and detailed information on CHARLS has been published [18].

Data were collected from CHARLS during 2011‐2020. All information related to diabetes, sarcopenia, and sleep disorders among participants aged ≥45 years could be downloaded from 2011, 2013, 2015, 2018, and 2020.

We selected participants according to the following steps and excluded based on the following criteria: (1) lack of age information or age<45 years in 2011; 2) missing height and weight measurements at baseline; (3) height and weight data beyond mean (3 SD); (4) missing gender information; (5) missing sarcopenia-related information; (6) missing FPG (fasting plasma glucose) information; (7) FPG <3.9 mmol/L, (8) no sleep status at baseline, (9) nightly sleep duration <6 or >8 hours; (10) questionnaire lacked diabetes self-report information, (11) normal FPG but choosing a self-reported diagnosis of “diabetes or high blood sugar” for the question “Have you been diagnosed with by a doctor?”.

Subsequently, all the participants selected at baseline were matched by ID to the database of 2013, 2015, 2018, and 2020, respectively; those without necessary information about sleep duration were excluded. Only the first record of occurrence of sleep disorder was retained, if one ID was found repeatedly across years.

### Measures

The measurements of exposure variables (ie, diabetes, sarcopenia), outcome variables (ie, sleep disorder, change of sleep duration or status), and covariates (ie, age, gender, education level, habitation, current marital status, current smoking, and drinking status) were respectively assessed across the cohort at each survey wave.

### Assessment of Diabetes

All participants were classified as having diabetes, prediabetes, and nondiabetes according to the Guidelines for the Prevention and Treatment of type 2 Diabetes Mellitus in China (2020 edition) [19]. Since only FPG in venous blood was measured in the CHARLS, the diagnosis in this study was based only on FPG values. A venous FPG value ≥7.0 mmol/L was classified as diabetes, FPG ≥6.1 mmol/L and <7.0 mmol/L as prediabetes, and FPG <6.0 mmol/L as nondiabetes. Although the baseline survey included the question “Have you been diagnosed with by a doctor?”, the participants answering “diabetes or high blood sugar, (including impaired glucose tolerance and elevated fasting blood sugar),” were excluded, as it was not possible to distinguish between diabetes and prediabetes.

### Assessment of Sarcopenia

The Asian Working Group for Sarcopenia: 2019 Consensus Update on Sarcopenia Diagnosis and Treatment (AWGS 2019) was used to assess the status of the sarcopenia and classify participants into sarcopenia, possible sarcopenia, and nonsarcopenia groups. The average of maximum values of handgrip strength was measured using either or both hands to evaluate the skeletal muscle strength. According to AWGS 2019, the threshold for low handgrip strength is <18 kg for women and <28 kg for men. The 5-time chair stand test was used to evaluate physical performance of the participants; a time ≥12 s of the 5-time chair stand test was defined as low physical performance. Individuals who could not complete the test were excluded. The muscle mass of the participants was evaluated using a previously validated formula [20]:


$$\begin{array}{l} ASM=0.193\times body\;weight\;(kg)+0.107\times \\ height\;(cm)-4.157\times sex\;(1=male,2=\\ female)–0.037\times age\;(years)-2.631 \end{array}$$

Following the estimation of appendicular skeletal muscle mass index (ASM) using the above formula, the appendicular skeletal muscle mass index (ASMI) was calculated using the ASM divided by the square of height in meters (ASMI = ASM/height^2^ m^2^). Therefore, the cut-off values for ASMI used in this study were based on the percentile value of the lowest 20% of the study population; low muscle mass was defined as ASMI <7.05 kg/m^2^ for men and <5.36 kg/m^2^ for women. Sarcopenia was diagnosed if muscle mass, muscle strength, or physical performance were low, and possible sarcopenia was diagnosed if muscle strength or physical performance was low.

### Covariates

Based on the literature review, some common factors correlated with sleep disorders, including demographic factors (eg, age and gender), area of residence (ie, city or rural), current marital status (ie, married or unmarried), and education level with a cut-off of junior high school graduation were selected as covariates to divide the participants into two groups. Health–related factors included current smoking and alcohol consumption status.

### Statistical Analysis

The categorical variables such as gender, education level, and habitation were expressed as percentages. The *χ*^2^ test was used for comparison between groups and Fisher exact test was used for analyses when the *χ*^2^ test was not appropriate. For quantitative indicators such as age, FPG, and muscle mass index, the mean (SD) was used for normally distributed variables. When the variables were not normally distributed, median with interquartile range was used calculated. The 2-tailed *t* test, ANOVA, or Kruskal-Wallis tests were used for comparison among groups, as appropriate. Kaplan-Meier survival analysis and log-rank test were used to compare the incidence of sleep disorders. Based on the different adjustment covariates, three Cox proportional hazards regression models were used to analyze the combined effect of diabetes and sarcopenia on sleep disorders. The restricted cubic spline (RCS) models were used to analyze the exposure-response and nonlinear relationships between FPG, muscle mass index, grip strength, 5-time chair stand test, and sleep disorders. Microsoft Excel (2021), SPSS software (version 27.0; IBM Corp) and R software (version 4.3.2; R Foundation for Statistical Computing) were used to conduct data collation, statistical testing, and RCS modelling respectively. A *P*<.05 was considered statistically significant.

### Ethical Considerations

In this study, all data were extracted from CHARLS, which received approval from Peking University Biomedical Ethics Committee (IRB00001052-11015) prior to conducting the original field investigation and all the participants had signed the informed consent form before enrollment. The secondary analysis was approved and anonymized data was used for analysis.

## Results

**Figure 1.**
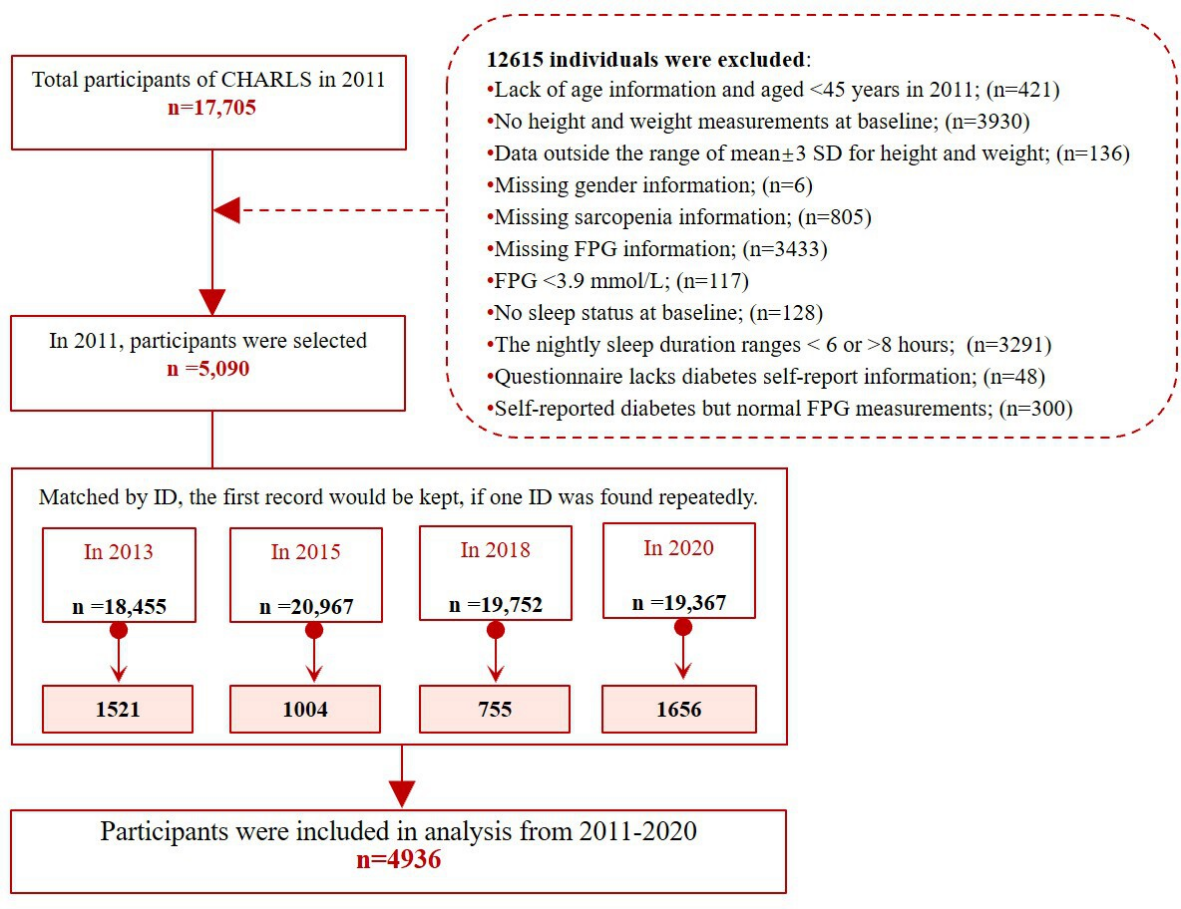
The flow chart of screening for enrolled individuals. CHARLS: China Health and Retirement Longitudinal Study; FPG: fasting plasma glucose.

A total of 17,705 participants were included at baseline. Of these, 12,615 were gradually excluded based on the exclusion criteria, and matched with follow-up data from 2013, 2015, 2018, and 2020 according to the participant ID. The first occurrence of sleep disorders was retained in the database. Finally, 4936 participants were included in the analysis (Figure 1).

A total of 4936 participants were selected and followed up on their sleep during 4 follow-up visits from 2011 to 2020, including 2427 male (49.2%) and 2509 female (50.8%) participants. Further comparison of the combination of diabetes and sarcopenia at different stages—NDNS, NDPS, NDS, PDNS, PDPS, PDS, DNS, DPS, and DS were statistically significant for age, sex, marital status, place of residence, education level, smoking, alcohol consumption, review status, weight, grip strength, skeletal muscle mass, 5-time chair stand test, and blood glucose levels *(*all *Ps*<.05; Table 1).

**Table 1.** Sociodemographic characteristics and critical variables of participants.

Variables	NDNS (Nondiabetes and Nonsarcopenia), n=2288	NDPS (Nondiabetes and Possible sarcopenia), n=841	NDS (Nondiabetes and Sarcopenia), n=324	PDNS (Prediabetes and Nonsarcopenia), n=599	PDPS (Prediabetes and Possible sarcopenia), n=230	PDS (Prediabetes and Sarcopenia), n=75	DNS (Diabetes and Nonsarcopenia), n=380	DPS (Diabetes and Possible sarcopenia), n=162	DS (Diabetes and Sarcopenia), n=37	H (*df*)/χ2 (*df*)	*P* value
Age (years), median (IQR)	55 (49-61)	58 (52-64)	68 (60-74)	57 (50-63)	60 (55-65)	68 (60-75)	57 (51-63)	59 (55-65)	70 (62-75)	528.833^a^ (8)	<.001
Gender, n (%)										37.412^b^ (8)	<.001
Male	1168 (51.0)	349 (41.5)	146 (45.1)	323 (53.9)	111 (48.3)	39 (52.0)	203 (53.4)	68 (42.0)	20 (54.1)		
Female	1120 (49.0)	492 (58.5)	178 (54.9)	276 (46.1)	119 (51.7)	36 (48.0)	177 (46.6)	94 (58.0)	17 (45.9)		
Marriage, n (%)											<.001^c^
Yes	2120 (92.7)	772 (91.8)	261 (80.6)	556 (92.8)	198 (86.1)	58 (77.3)	355 (93.4)	143 (88.3)	28 (75.7)		
No	168 (7.3)	69 (8.2)	63 (19.4)	43 (7.2)	32 (13.9)	17 (22.7)	25 (6.6)	19 (11.7)	9 (24.3)		
Habitation, n (%)											<.001^c^
City	2098 (91.7)	796 (94.6)	314 (96.9)	543 (90.7)	205 (89.1)	73 (97.3)	341 (90.0)	149 (92.0)	35 (94.6)		
Rural	190 (8.3)	45 (5.4)	10 (3.1)	56 (9.3)	25 (10.9)	2 (2.7)	38 (10.0)	13 (8.0)	2 (5.4)		
Education, n (%)										169.139^b^ (8)	<.001
Above of junior high school graduation	946 (41.3)	225 (26.8)	45 (13.9)	235 (39.2)	68 (29.6)	12 (16.0)	159 (42.0)	40 (24.7)	6 (16.2)		
Others	1342 (58.7)	616 (73.2)	278 (86.1)	364 (60.8)	162 (70.4)	63 (84.0)	220 (58.0)	122 (75.3)	31 (83.8)		
Smoking	935 (40.9)	298 (35.5)	117 (36.1)	277 (46.2)	90 (39.1)	38 (50.7)	160 (42.1)	62 (38.3)	17 (45.9)	24.306^b^ (8)	.002
Drinking	864 (37.8)	234 (27.8)	91 (28.1)	248 (41.4)	72 (31.3)	24 (32.0)	165 (43.4)	41 (25.3)	9 (24.3)		<.001
Height(cm), median (IQR)	159.5(153.4-165.5)	157.8(152.4-163.9)	154.9 (146.8-161.0)	159.5 (153.3-165.8)	158.5 (153.5-164.8)	154.0 (146.0-154.0)	159.5 (152.9-165.3)	156.0(151.7-162.3)	157.0(151.0-163.2)	138.643^a^ (8)	<.001
Weight (kg), median (IQR)	58.7 (52.1-66.2)	60.2 (55.1-67.0)	45.6 (42.2-49.3)	61.0 (54.6-69.4)	63.3 (57.3-69.2)	46.6 (42.3-50.3)	61.6 (53.3-69.9)	61.4 (55.8-68.7)	46.4 (42.5-51.2)	878.573^a^ (8)	<.001
Handgrip strength (kg), median (IQR)	34.0 (28.0-41.0)	26.5 (20.0-34.4)	22.5 (17.0-27.7)	34.0 (28.5-41.6)	27.5 (19.3-35.2)	22.6(17.5-29.5)	34.0 (28.1-41.3)	25.0 (18.5-32.5)	21.6 (17.0-29.3)	818.091^a^ (8)	<.001
ASM^d^ (kg/m^2^), median (IQR)	17.8 (14.3-20.7)	16.7 (14.4-20.3)	12.9 (10.6-17.1)	18.1 (14.9-21.5)	18.2 (14.9-21.1)	13.9 (10.6-17.7)	18.3 (14.6-21.3)	16.7 (14.4-20.4)	14.7 (11.6-17.3)	343.836^a^ (8)	<.001
5-time chair stand test (s), median (IQR)	8.6 (7.2-10.2)	13.5 (12.3-15.4)	13.3 (11.2-15.5)	8.9 (7.4-10.3)	13.5 (12.2-16.3)	13.3 (11.3-16.3)	9.0 (7.5-10.3)	13.0 (12.3-15.4)	13.6 (11.2-17.3)	2336.735^a^ (8)	<.001
FPG^e^ (mmol/L), median (IQR)	5.5 (5.2-5.8)	5.5 (5.1-5.8)	5.4 (5.1-5.7)	6.4 (6.3-6.6)	6.4 (6.2-6.6)	6.4 (6.2-6.6)	8.0 (7.3-9.5)	8.0 (7.4-9.4)	7.8 (7.3-8.9)	3208.235^a^ (8)	<.001

a Kruskal-Wallis H test.

b *χ*2: chi-square statistic.

c Fisher’s exact test.

d ASM: appendicular skeletal muscle mass.

e FPG: fasting plasma glucose.

**Figure 2.**
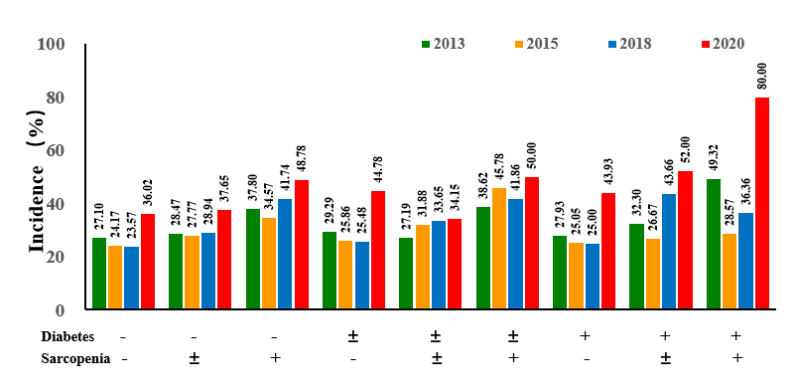
The incidence of sleep disorders of the co-exposure of diabetes and sarcopenia at different stages. In the diabetes group, (+) represents diabetes, (±) represents prediabetes, and (-) depicts nondiabetes. In the sarcopenia group, (+) represents sarcopenia, (±) indicates possible sarcopenia, and (-) depicts nonsarcopenia.

**Figure 3.**
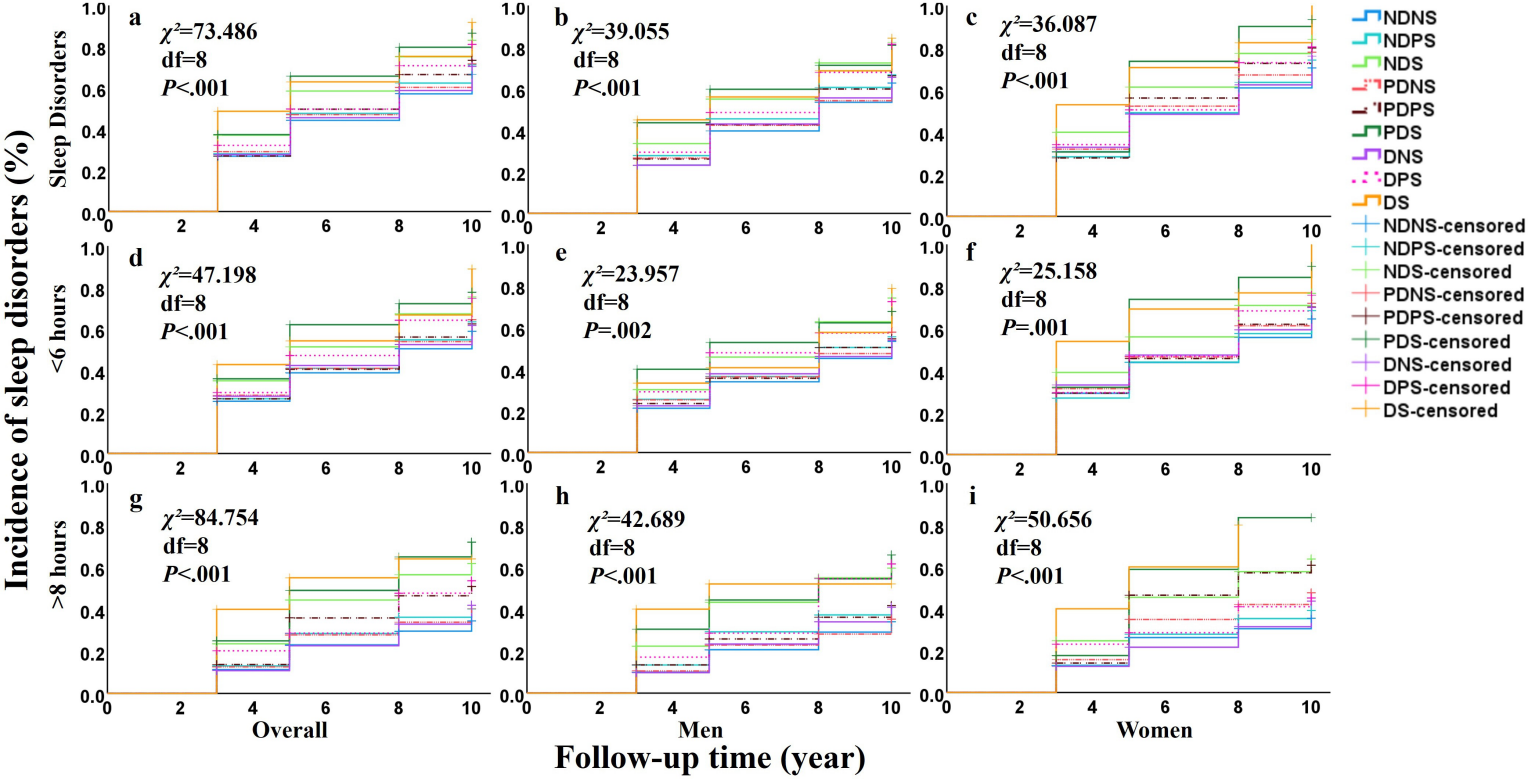
Incidence of sleep disorders related to co-exposure of diabetes and sarcopenia among groups. Follow-up time depicts the number of years of follow-up in 2013, 2015, 2018, 2020, respectively. *χ*^2^ indicates the statistic from the log-rank test. NDNS: Nondiabetes and Nonsarcopenia, NDPS: Nondiabetes and Possible Sarcopenia, NDS: Nondiabetes and Sarcopenia, PDNS: Prediabetes and Nonsarcopenia, PDPS: Prediabetes and Possible Sarcopenia, PDS: Prediabetes and Sarcopenia, DNS: Diabetes and Nonsarcopenia, DPS: Diabetes and Possible Sarcopenia, DS: Diabetes and Sarcopenia; SD: sleep disorders (including sleep duration <6h and >8h); <6 hours: sleep duration at the final follow-up was <6 hours; >8 hours: sleep duration at the final follow-up was >8 hours.

Based on survival analysis, it was observed that with the change in follow-up time, there was a significant difference in the incidence of sleep disorders among all groups (*χ^2_8_^*=73.486, *P*<.001; Figure 2). In 2020, the DS group had the highest incidence of sleep disorders (80. and the incidence in the DPS, PDS and NDS groups reached 52.00%, 50.00%, and 48.78%, respectively; whereas, the incidence of sleep disorders was the lowest in the NDNS group (36.02%). Furthermore, the incidence of sleep disorders in the DS group was 49.32%, 28.57%, 36.36%, and 80.00% in 2013, 2015, 2018, and 2020, respectively, showing a significantly increasing trend. Significant differences were also observed between men and women, and the incidence of short sleep duration (<6 hours) and long sleep duration (>8 hours), as shown in Figure 3.

According to Cox regression analysis, there was a significant association between the combined status of diabetes mellitus, sarcopenia, and sleep disorders. In Model 1, no covariates were adjusted, compared with the NDNS group, the risk of sleep disorders was increased in the DS group (HR 1.707, 95% CI 1.196‐2.437, *P*=.003), PDS (HR 1.599, 95% CI 1.235‐2.071, *P*<.001), NDS (HR 1.465, 95% CI 1.282‐1.674, *P*<.001), and DPS (HR 1.318, 95% CI 1.097‐1.583, *P*=.003). The risk was increased but not statistically significant in the PDPS group. After adjusting for the variables (Model 3), the risk of sleep disorders in the DS group (HR 1.515, 95% CI 1.059‐2.167; *P*=.02) was still statistically significant, and the risk in the PDS (HR 1.423, 95% CI 1.096‐1.847; *P*=.008) and NDS (HR 1.279,95% CI 1.113‐1.468; *P*<.001) groups were significantly higher than that in the NDNS group (Table 2).

**Table 2.** Association between sleep disorders and co-exposure to diabetes and sarcopenia at different status. Symbols indicate existing condition: diabetes (+), nondiabetes (–), prediabetes (±) in the diabetes group, and sarcopenia (+), possible sarcopenia (–), and no sarcopenia (±) in the sarcopenia group.

Group		Model 1^a^, HR^b^ (95% CI)	Model 2^c^, HR (95% CI)	Model 3^d^, HR (95% CI)
Diabetes	Sarcopenia			
Sleep disorders^e^				
-	-	Reference	Reference	Reference
-	±	1.105 (1.003-1.218)	1.058 (0.959-1.167)	1.033 (0.936-1.139)
-	＋	1.465 (1.282-1.674)	1.344 (1.171-1.541)	1.279 (1.113-1.468)
±	-	1.107 (0.992-1.235)	1.100 (0.986-1.277)	1.098 (0.984-1.225)
±	±	1.160 (0.987-1.365)	1.114 (0.946-1.311)	1.110 (0.943-1.307)
±	+	1.599 (1.235-2.071)	1.501 (1.157-1.946)	1.423 (1.096-1.847)
+	-	1.067 (0.935-1.219)	1.061 (0.929-1.212)	1.070 (0.936-1.223)
+	±	1.318 (1.097-1.583)	1.251 (1.040-1.504)	1.228 (1.021-1.477)
+	+	1.707 (1.196-2.437)	1.594 (1.115-2.279)	1.515 (1.059-2.167)
Sleep duration <6 hours				
-	-	Reference	Reference	Reference
**-**	±	1.100 (0.981-1.234)	1.046 (0.932-1.174)	1.023 (0.911-1.149)
-	＋	1.455 (1.233-1.716)	1.343 (1.134-1.591)	1.286 (1.084-1.525)
±	-	1.120 (0.985-1.273)	1.120 (0.985-1.273)	1.118 (0.983-1.271)
±	±	1.086 (0.884-1.334)	1.059 (0.861-1.302)	1.061 (0.863-1.305)
±	+	1.596 (1.139-2.235)	1.503 (1.071-2.110)	1.417 (1.008-1.992)
+	-	1.073 (0.915-1.258)	1.070 (0.913-1.255)	1.075 (0.916-1.261)
+	±	1.366 (1.099-1.697)	1.287 (1.035-1.601)	1.260 (1.012-1.568)
+	+	1.713 (1.123-2.612)	1.623 (1.062-2.481)	1.546 (1.010-2.365)
Sleep duration >8 hours				
-	-	Reference	Reference	Reference
-	±	1.208 (1.007-1.450)	1.137 (0.946-1.367)	1.073 (0.892-1.290)
-	＋	2.132 (1.696-2.681)	1.791 (1.415-2.268)	1.601 (1.262-2.030)
±	-	1.197 (0.972-1.474)	1.173 (0.952-1.445)	1.160 (0.942-1.429)
±	±	1.594 (1.222-2.080)	1.465 (1.121-1.914)	1.429 (1.093-1.869)
±	+	2.545 (1.699-3.814)	2.256 (1.502-3.389)	2.033 (1.351-3.058)
+	-	1.198 (0.941-1.525)	1.162 (0.913-1.480)	1.195 (0.937-1.524)
+	±	1.665 (1.184-2.343)	1.516 (1.076-2.136)	1.502 (1.065-2.118)
+	+	2.739 (1.414-5.306)	2.340 (1.205-4.543)	1.970 (1.011-3.840)
Decreased sleep duration^f^				
-	-	Reference	Reference	Reference
-	±	1.122 (1.014-1.242)	1.073 (0.969-1.189)	1.052 (0.949-1.166)
-	＋	1.368 (1.175-1.593)	1.274 (1.090-1.490)	1.224 (1.046-1.433)
±	-	1.076 (0.958-1.208)	1.069 (0.952-1.201)	1.067 (0.950-1.199)
±	±	1.121 (0.933-1.349)	1.091 (0.907-1.313)	1.101 (0.914-1.325)
±	+	1.735 (1.261-2.386)	1.617 (1.174-2.229)	1.525 (1.105-2.104)
+	-	1.063 (0.923-1.224)	1.058 (0.918-1.219)	1.069 (0.928-1.232)
+	±	1.251 (1.018-1.537)	1.188 (0.966-1.461)	1.167 (0.948-1.435)
+	+	1.546 (1.015-2.357)	1.452 (0.951-2.216)	1.391 (0.910-2.124)
Increased sleep duration^g^				
-	-	Reference	Reference	Reference
-	±	1.253 (1.078-1.457)	1.212 (1.042-1.409)	1.167 (1.002-1.358)
-	＋	1.674 (1.363-2.056)	1.468 (1.188-1.814)	1.385 (1.119-1.713)
±	-	1.115 (0.937-1.325)	1.099 (0.924-1.308)	1.081 (0.908-1.286)
±	±	1.377 (1.094-1.733)	1.295 (1.028-1.631)	1.259 (0.998-1.588)
±	+	1.952 (1.367-2.787)	1.815 (1.270-2.595)	1.697 (1.185-2.429)
+	-	1.104 (0.901-1.353)	1.078 (0.879-1.322)	1.088 (0.886-1.335)
+	±	1.270 (0.941-1.714)	1.196 (0.885-1.616)	1.201 (0.888-1.624)
+	+	2.067 (1.167-3.662)	1.852 (1.043-3.288)	1.641 (0.920-2.924)

a Model 1 represents no covariates were adjusted.

b HR: hazard ratio.

c Model 2 represents adjusting for gender and age.

d Model 3 represents adjustment for gender, age, education, current marital status and habitation, smoking, and drinking.

e Sleep disorders including sleep duration <6 hours and >8 hours.

f Decreased indicates difference of sleep duration between baseline and follow-up <0.

g Increased indicates the difference of sleep duration between baseline and follow-up >0.

After a stratified analysis of sleep duration (ie, <6 hours and >8 hours), the risk of short sleep duration (< 6 hours) was significantly increased in the DS group without adjusting for variables (Model 1: HR 1.713, 95% CI 1.123‐2.612) and remained statistically significant after adjusting for variables (Model 3: HR 1.546, 95% CI 1.010‐2.365). The risk of long sleep duration (> 8 hours) was significantly increased in the DS group without adjusting for variables (Model 1: HR 2.739, 95% CI 1.414‐5.306), which remained statistically significant after adjusting for variables (Model 3: HR 1.970, 95% CI 1.011‐3.840; Table 2).

To analyze the changes in sleep duration, we stratified participants based on the difference in sleep duration between the final follow-up and baseline. If the difference was <0, it was defined as “Decrease,” while a difference >0, was defined as “Increase in sleep duration.” Cox regression analysis revealed that the risk of “Decrease” in sleep duration in the NDS group without adjusting for the variable (Model 1) is significantly higher (HR 1.735, 95% CI 1.261‐2.386), and this remained statistically significant after adjusting for covariates (Model 3: (HR 1.525, 95% CI 1.105‐2.104). After adjusting for covariates, the risk of “Increase” in the PDS (Model 3: HR 1.697, 95% CI 1.185‐2.429) and NDS (Model 3: HR 1.385, 95% CI 1.119‐1.713) groups also increased significantly (Table 2).

**Figure 4.**
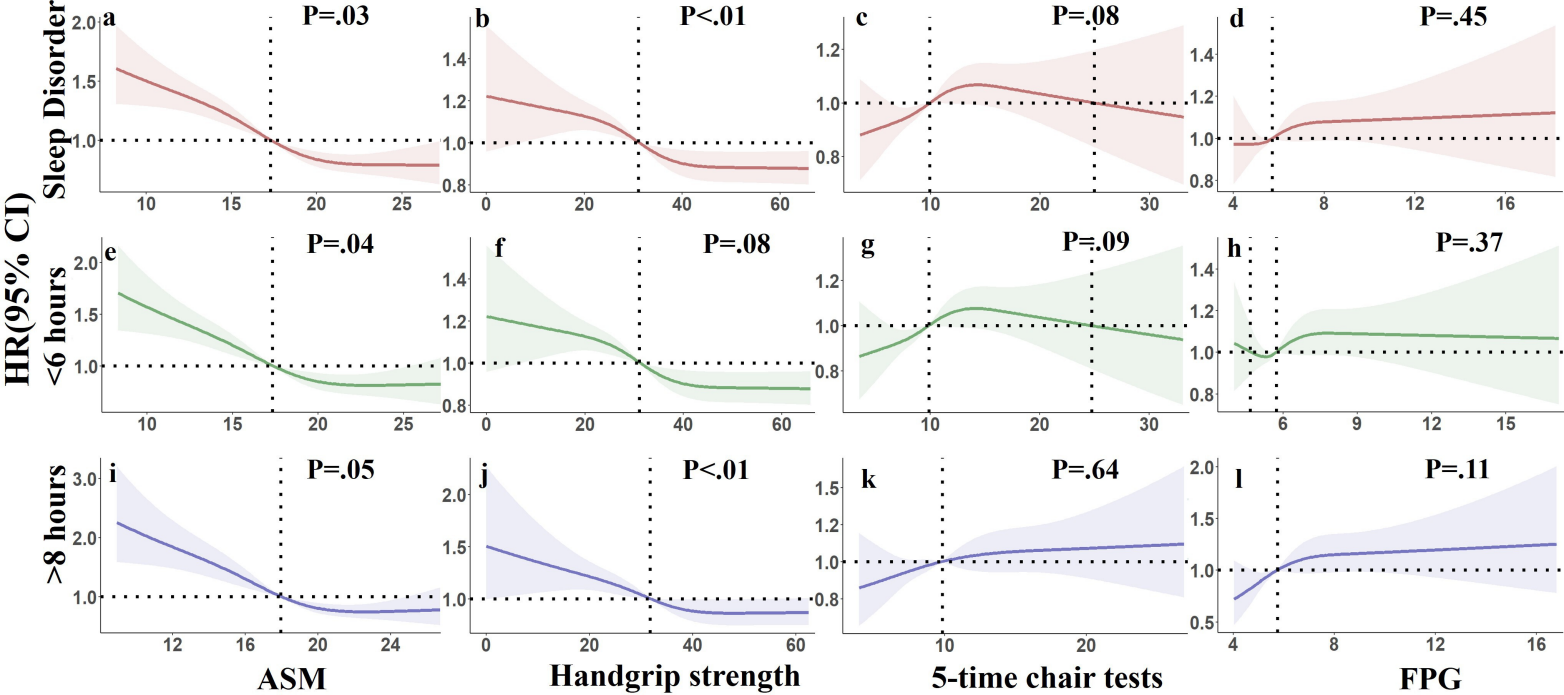
Exposure-response relationship among diabetes, sarcopenia, and sleep disorders.. The restricted cubic spline (RCS) was used to model expose-response curves after adjusting for factors including gender, age, education, current marital status, and habitation, smoking, and drinking. ASM: appendicular skeletal muscle mass; FPG: fasting plasma glucose; HR: hazard ratio; SD: sleep disorders; <6 h: sleep duration was <6 hours; >8 h: sleep duration was >8 hours.

To further assess the relationship between diabetes, sarcopenia, and sleep disorders, the RCS model was used to describe important factors related to sarcopenia—ASM, handgrip strength, 5-time chair stand test performance—and the exposure response relationship between FPG levels, and sleep disorders. Figure 4 illustrates these association of sleep disorders with ASM (*χ^2^*=45.04, *P*<.001, *P* for nonlinearity=.0284), handgrip strength (*χ^2^*=16.62, *P*<.008, *P* for nonlinearity<.0017), and 5-time chair stand tests (*χ^2^*=8.78, *P*=.03, *P* for nonlinearity=.0806). A nonlinear correlation was observed between sleep disorders and both ASM and handgrip strength. The relationship between FPG and long sleep duration (>8 hours) was also significant (*χ^2^*=9.44, *P*=.02, *P* for nonlinearity=.10731).

## Discussion

With increasing age, the issue of sleep disorders in older adults is becoming more severe [21]. Common chronic conditions including diabetes mellitus and sarcopenia which are highly prevalent could affect sleep quality. In particular, prediabetes and possible sarcopenia are now being diagnosed at an early age. If this status is not addressed during reversible stages, the risk of sleep disorders in adults aged ≥45 years is even more severe. Although studies have reported a relationship between diabetes [22] or sarcopenia [23] and sleep disorders, there is a lack of studies on the relationship between co-exposure at varying stages of diabetes and sarcopenia. This study found that participants with diabetes and sarcopenia had the highest risk of sleep disorders; groups with prediabetes and sarcopenia, nondiabetes with sarcopenia, and diabetes with possible sarcopenia had a significantly higher risk of sleep disorders. Additionally, there was an exposure-response relationship between ASM, handgrip strength, 5-time chair test, FPG levels, and sleep disorders. These findings can provide a reference for the prevention of sleep disorders in adults aged ≥45 years and the development of early screening and personalized intervention.

Diabetes, as a chronic disease with high incidence in older adults, is an important factor contributing to sarcopenia [24] and sleep disorders [25]. Our findings showed that participant groups with both diabetes and prediabetes have an increased risk of sleep disorders. This may be explained by the following mechanisms. On one hand, diabetes can affect the function of the hypothalamic-pituitary-adrenal axis, resulting in abnormal secretion of hormones such as cortisol [26]. The rhythm of these hormones can affect the sleep-wake cycle, leading to the sleep disorders [27]. On the other hand, hyperglycemia may lead to the secretion of melatonin, which plays a key role in regulating sleep rhythms [28]. Its altered secretion can lead to difficulty falling asleep and poor sleep quality [29]. In addition, patients with sarcopenia are prone to muscle fatigue and damage due to the loss of mass and strength of several muscles [30]. During sleep, changes in body posture may strain already fragile muscles, causing pain and discomfort, resulting in sleep disruption. Muscle strength in the chest and abdomen decreases in patients with sarcopenia, resulting in decreased respiratory function [31]. During sleep, weakness in respiratory muscles may cause poor breathing, snoring and even apnea, thus interfering with sleep [32]. It is also important to note that diabetes can cause sarcopenia. Long-term hyperglycemia may lead to peripheral neuropathy [33], causing a decrease in muscle blood supply, affecting the nutrition and innervation of nerves to muscles [34], which in turn leads to muscle waste atrophy. Patients with diabetes and prediabetes have widespread insulin resistance, which blocks insulin signaling pathways such as PI3K/Ak [35] and Ras-MAPK [36], thus cannot effectively promote the uptake of amino acids by muscle cells, thereby inhibiting the synthesis of muscle proteins; this results in a decrease in muscle mass. These mechanisms reveal the relationship between FPG, ASM, handgrip strength, 5-time chair stand test, and sleep disorders, as identified by the RCS model.

This study found that participants with comorbid diabetes and sarcopenia had the highest risk of sleep disorders, and the groups with prediabetes and sarcopenia, nondiabetes and sarcopenia, and diabetes with possible sarcopenia also had a significantly higher risk of sleep disorders. The potential mechanisms of sleep disorders caused by the comorbidity of diabetes and sarcopenia may be related to the following: (1) patients with diabetes already have abnormal blood sugar regulation; sarcopenia will further affect the uptake and utilization of glucose by muscles, resulting in more frequent and difficult-to-control blood sugar fluctuations [37]. This may result in the superposition of metabolic disorders, triggering energy metabolism imbalance [38], making the body unable to obtain enough energy at night to maintain normal physiological functions and sleep status. (2) Neuropathy caused by diabetes, including damage to peripheral and autonomic nerves can affect the sensory and motor functions of muscles [39]. Sarcopenia aggravates muscle weakness and atrophy, further impairing the function of the neuromuscular junction [40]. This neuromuscular dysfunction can lead to nocturnal muscle twitches, spasms, or pain that interfere with sleep. Autonomic dysfunction is common in both diabetes and sarcopenia; it affects the regulation of the cardiovascular and respiratory systems, resulting in abnormal fluctuations in blood pressure and heart rate at night, poor breathing, and increases the risk of sleep disruption [41]. Both diabetes and sarcopenia are associated with abnormal hormone secretion [42], which may lead to reduced secretion of testosterone, estrogen, and insulin-like growth factor 1 (IGF-1) among others. Significant gender differences were observed in our study, which may further support this phenomenon. Furthermore, diabetes and sarcopenia are both accompanied by chronic inflammation and increased levels of oxidative stress [43]. Tumor necrosis factor α (TNF-α), interleukin 6 (IL-6) promote muscle catabolism and may affect the central nervous system function, resulting in disorders in sleep regulation centers. Reactive oxygen species produced during oxidative stress can damage both neural and muscular systems, aggravate physical discomfort, and then affect sleep [44].

Our findings have several implications. First, special attention should be paid to adults aged ≥45 years with sleep disorders. To improve sleep duration and quality, it is necessary to effectively manage diabetes, perform physical exercises to improve muscle mass and function, and continuously monitor sleep quality. Second, for people with prediabetes or possible sarcopenia, early intervention should be carried out to prevent the occurrence of diabetes or sarcopenia, which can effectively reduce the risk of sleep disorders. Third, the community should build appropriate sports facilities and provide an environment that promotes physical exercise to improve the health of residents. In addition, regular screening for diabetes and sarcopenia should be implemented to detect patients with diabetes and sarcopenia at an early stage, and comprehensive interventions should be actively used to prevent sleep disorders.

The advantages of this study include the following aspects. It is a large-scale, nationally representative cohort study of community residents, using survival analysis and Cox regression models to explore the co-exposure of diabetes and sarcopenia—as well as the impact of prediabetes with latent risk and possible sarcopenia—on sleep duration and sleep status. However, there are also a few limitations within our study: First, due to limitations in the CHARLS data, we could not fully explore the relationship between diabetes status, progression trends in sarcopenia from 2011 to 2020, and specific types of sleep disorders. Our analysis was limited to examining the impact of diabetes and sarcopenia status on sleep disorders in 2011; the relationship between the factors affecting progression and sleep disorders is complex. Concurrently, due to limited data, we included a smaller number of participants aged ≥75 years in our study, and the differences in population distribution across age groups may limit the extrapolation of our findings. Second, prediabetes is an intermediate stage that can revert toward normoglycemia and diabetes. Therefore, our study only analyzed the effect of prediabetes exposure at baseline on sleep disorders. Since the CHARLS did not provide data on FPG levels for the four follow-up waves , it was impossible to explore the role of changes in prediabetes status on sleep disorders. However, our findings still suggest that patients with prediabetes were at an increased risk of sleep disorders. Therefore, family-based and both individual health education [45], and community-level behavioral interventions play an important role in the prevention of sleep disorders [46]. Fourth, there is a mutual relationship among diabetes, sarcopenia, and sleep disorders. Although both diabetes and sarcopenia can lead to sleep disorders, the interaction between diabetes and sarcopenia on sleep disorders could not be analyzed due to lack of sufficient data. Fifth, this study found a potential relationship between the co-exposure to various diabetes and sarcopenia statuses, and sleep disorders. Diabetes and sarcopenia may have a synergistic effect on the occurrence of sleep disorders; however, causality cannot be based solely on our findings. Future research should focus on the progression and changes of diabetes, sarcopenia, and sleep disorders, including sleep efficiency [47] to explore the relationship among them.

In conclusion, this study found that diabetes combined with sarcopenia in adults aged 45 years and older may increase the risk of sleep disorders. To reduce this risk, it is essential to implement effective disease management for both diabetes and sarcopenia, and to further evaluate joint intervention measures for individuals with prediabetes or possible sarcopenia.

## Abbreviations

ASM: appendicular skeletal muscle mass
ASMI: appendicular skeletal muscle mass index
AWGS: Asian Working Group for Sarcopenia: 2019 Consensus Update on Sarcopenia Diagnosis and Treatment
CHARLS: China Health and Retirement Longitudinal Study
FPG: fasting plasma glucose
HR: hazard ratio
RCS: restricted cubic spline

## References

[1] Canever JB, Zurman G, Vogel F (2024). Worldwide prevalence of sleep problems in community-dwelling older adults: a systematic review and meta-analysis. Sleep Med.

[2] Yue L, Cui N, Liu Z, Jia C, Liu X (2022). Patterns of sleep problems and internalizing and externalizing problems among Chinese adolescents: a latent class analysis. Sleep Med.

[3] Liu SH, Lin FJ, Kao YH (2024). Chronic partial sleep deprivation increased the incidence of atrial fibrillation by promoting pulmonary vein and atrial arrhythmogenesis in a rodent model. Int J Mol Sci.

[4] Robbins R, Quan SF, Weaver MD, Bormes G, Barger LK, Czeisler CA (2021). Examining sleep deficiency and disturbance and their risk for incident dementia and all-cause mortality in older adults across 5 years in the United States. Aging (Albany NY).

[5] Haddadi A, Matinnia N, Yazdi-Ravandi S (2024). The relationship between corona disease anxiety and sleep disturbances and suicidal ideation in medical staff: The mediating role of resiliency and cognitive flexibility: a cross-sectional study. Health Sci Rep.

[6] Wang J, Gueye-Ndiaye S, Castro-Diehl C (2024). Associations between indoor fine particulate matter (PM_2.5_) and sleep-disordered breathing in an urban sample of school-aged children. Sleep Health.

[7] Zheng YB, Huang YT, Gong YM (2024). Association of lifestyle with sleep health in general population in China: a cross-sectional study. Transl Psychiatry.

[8] Algethami A, Alfahmi FK, Alhusayni MA (2024). Evaluation of sleep quality among people living with type 2 diabetes mellitus in Taif, Saudi Arabia: a cross-sectional study. Cureus.

[9] Liu K, Luo J, Chen Y (2024). Association between sarcopenia and sleep disorders: a cross-sectional population based study. Front Nutr.

[10] Diabetes around the world in 2021. International Diabetes Federation.

[11] Lingling G, Lin Y, Jiao Y (2019). Prevalence and influencing factors of diabetes for the 45 years and older population in China. Modern Preventive Medicine.

[12] Petermann-Rocha F, Balntzi V, Gray SR (2022). Global prevalence of sarcopenia and severe sarcopenia: a systematic review and meta-analysis. J Cachexia Sarcopenia Muscle.

[13] Woo J, Lehrer HM, Tabibi D, Cebulske L, Tanaka H, Steinhardt M (2024). The association of multidimensional sleep health with hba1c and depressive symptoms in African American Adults with type 2 diabetes. Psychosom Med.

[14] Taimah M, Ahmad A, Al-Houqani M (2024). Association between obstructive sleep apnea risk and type 2 diabetes among Emirati adults: results from the UAE healthy future study. Front Endocrinol (Lausanne).

[15] Ni MH, Yang YS, Huang JY, Lo SC, Huang CN, Kornelius E (2024). The association of depression and sleep disorders in patients with type 1 diabetes in Taiwan. Medicine (Baltimore).

[16] Lv X, Peng W, Jia B, Lin P, Yang Z (2024). Longitudinal association of sleep duration with possible sarcopenia: evidence from CHARLS. BMJ Open.

[17] Ida S, Kaneko R, Nagata H (2019). Association between sarcopenia and sleep disorder in older patients with diabetes. Geriatr Gerontol Int.

[18] Zhao Y, Strauss J, Yang G, Giles J (2013). 2011-2012 national baseline user’s guide [chinese]. China Health and Retirement Longitudinal Study.

[19] Zhu D, Chinese Diabetes Society (2021). Guideline for the prevention and treatment of type 2 diabetes mellitus in China (2020 edition) [Chinese]. Chin J Diabetes Mellitus.

[20] Wen X, Wang M, Jiang CM, Zhang YM (2011). Anthropometric equation for estimation of appendicular skeletal muscle mass in Chinese adults. Asia Pac J Clin Nutr.

[21] Delbari A, Ahmadi F, Zar A, Zandvakili A, Sadeghipour HR, Sims J (2024). Living in urban or rural environments affect the sleep quality of the elderly in Bushehr (Southern Iran): emphasizing the active and inactive of the elderly. BMC Public Health.

[22] Tenda ED, Henrina J, Cha JH (2024). Obstructive sleep apnea: overlooked comorbidity in patients with diabetes. World J Diabetes.

[23] Smith L, Shin JI, Veronese N (2022). Sleep duration and sarcopenia in adults aged ≥ 65 years from low and middle-income countries. Aging Clin Exp Res.

[24] Chowdhury SR, Chakrabarti A, Datta PK (2023). Sarcopenia in patients with diabetes mellitus, an overlooked perioperative condition. Br J Anaesth.

[25] Kuroda H, Yeung SLA, Fujii R, Iwagami M, Goto A (2025). Investigating the non-linear association between sleep duration and type 2 diabetes: conventional and Mendelian randomization analyses from the UK Biobank. J Diabetes Investig.

[26] Marissal-Arvy N, Moisan MP (2022). Diabetes and associated cognitive disorders: role of the hypothalamic-pituitary adrenal axis. Metabol Open.

[27] Ba-Ali S, Brøndsted AE, Andersen HU, Sander B, Jennum PJ, Lund-Andersen H (2019). Assessment of diurnal melatonin, cortisol, activity, and sleep-wake cycle in patients with and without diabetic retinopathy. Sleep Med.

[28] Onaolapo AY, Adebisi EO, Adeleye AE, Olofinnade AT, Onaolapo OJ (2020). Dietary melatonin protects against behavioural, metabolic, oxidative, and organ morphological changes in mice that are fed high-fat, high-sugar diet. Endocr Metab Immune Disord Drug Targets.

[29] van Maanen A, Meijer AM, Smits MG, van der Heijden KB, Oort FJ (2017). Effects of melatonin and bright light treatment in childhood chronic sleep onset insomnia with late melatonin onset: a randomized controlled study. Sleep.

[30] Zhang Y, Chen X, Hou L (2020). Prevalence and risk factors governing the loss of muscle function in elderly sarcopenia patients: a longitudinal study in China with 4 years of follow-up. J Nutr Health Aging.

[31] Nagano A, Wakabayashi H, Maeda K (2021). Respiratory sarcopenia and sarcopenic respiratory disability: concepts, diagnosis, and treatment. J Nutr Health Aging.

[32] Peng X, Zhou R, Liu C, Chen X, Zhu T, Chen G (2024). Abnormal sleep duration is associated with sarcopenia in older Chinese people: A large retrospective cross-sectional study. Open Med (Wars).

[33] Albariqi MM, Versteeg S, Brakkee EM (2023). Human IAPP is a contributor to painful diabetic peripheral neuropathy. J Clin Invest.

[34] van Sloten TT, Savelberg HHCM, Duimel-Peeters IGP (2011). Peripheral neuropathy, decreased muscle strength and obesity are strongly associated with walking in persons with type 2 diabetes without manifest mobility limitations. Diabetes Res Clin Pract.

[35] Feng Y, Ren Y, Zhang X (2024). Metabolites of traditional Chinese medicine targeting PI3K/AKT signaling pathway for hypoglycemic effect in type 2 diabetes. Front Pharmacol.

[36] Kasowanjete P, Abrahamse H, Houreld NN (2023). Photobiomodulation at 660 nm stimulates in vitro diabetic wound healing via the Ras/MAPK Pathway. Cells.

[37] Norlin S, Axelsson J, Ericsson M, Edlund H (2023). O304 ameliorates hyperglycemia in mice by dually promoting muscle glucose effectiveness and preserving β-cell function. Commun Biol.

[38] Al-Sayyar A, Hammad MM, Williams MR, Al-Onaizi M, Abubaker J, Alzaid F (2023). Neurotransmitters in type 2 diabetes and the control of systemic and central energy balance. Metabolites.

[39] Zilliox LA (2021). Diabetes and peripheral nerve disease. Clin Geriatr Med.

[40] Miao Y, Xie L, Song J (2024). Unraveling the causes of sarcopenia: roles of neuromuscular junction impairment and mitochondrial dysfunction. Physiol Rep.

[41] Mizukami K (2023). Autonomic dysfunction in dementia with Lewy bodies: focusing on cardiovascular and respiratory dysfunction. PCN Rep.

[42] He Y, Fu Q, Sun M (2022). Phosphoproteome reveals molecular mechanisms of aberrant rhythm in neurotransmitter-mediated islet hormone secretion in diabetic mice. Clin Transl Med.

[43] Muvhulawa N, Mazibuko-Mbeje SE, Ndwandwe D (2023). Sarcopenia in a type 2 diabetic state: reviewing literature on the pathological consequences of oxidative stress and inflammation beyond the neutralizing effect of intracellular antioxidants. Life Sci.

[44] Singh G, Singh K, Sinha RA, Singh A, Kumar A (2024). Japanese encephalitis virus infection causes reactive oxygen species‐mediated skeletal muscle damage. Eur J of Neuroscience.

[45] Amerzadeh M, Shafiei Kisomi Z, Senmar M, Khatooni M, Hosseinkhani Z, Bahrami M (2024). Self-care behaviors, medication adherence status, and associated factors among elderly individuals with type 2 diabetes. Sci Rep.

[46] Van Ancum JM, Meskers CGM, Reijnierse EM (2020). Lack of knowledge contrasts the willingness to counteract sarcopenia among community-dwelling adults. J Aging Health.

[47] Sakal C, Li T, Li J, Yang C, Li X (2024). Association between sleep efficiency variability and cognition among older adults: cross-sectional accelerometer study. JMIR Aging.

